# Cognitive Biases toward Internet Game-Related Pictures and Executive Deficits in Individuals with an Internet Game Addiction

**DOI:** 10.1371/journal.pone.0048961

**Published:** 2012-11-14

**Authors:** Zhenhe Zhou, Guozhen Yuan, Jianjun Yao

**Affiliations:** Department of Psychology, Wuxi Mental Health Center of Nanjing Medical University, Wuxi City, Jiangsu Province, China; Catholic University of Sacred Heart of Rome, Italy

## Abstract

**Background:**

The cue-related go/no-go switching task provides an experimental approach to study individual’s flexibility in changing situations. Because Internet addiction disorder (IAD) belongs to the compulsive-impulsive spectrum of disorders, it should present cognitive bias and executive functioning deficit characteristics of some of these types of disorders. Until now, no studies have been reported on cognitive bias and executive function involving mental flexibility and response inhibition in IAD.

**Methodology/Principal Findings:**

A total of 46 subjects who met the criteria of the modified Young’s Diagnostic Questionnaire for Internet addiction (YDQ) were recruited as an Internet game addiction (IGA) group, along with 46 healthy control individuals. All participants performed the Internet game-shifting task. Using hit rate, RT, *d′* and C as the dependent measures, a three-way ANOVA (group × target × condition) was performed. For hit rate, a significant effect of group, type of target and condition were found. The group–target interaction effect was significant. For RT, significant effects were revealed for group and type of target. The group–target interaction effect was significant. Comparisons of the means revealed that the slowing down of IGA relative to NIA was more pronounced when the target stimuli were neutral as opposed to Internet game-related pictures. In addition, the group–condition interaction effect was significant. For *d′*, significant effects of group, type of target and condition were found. The group–target interaction effect was significant. For C, the type of target produced a significant effect. There was a positive correlation between the length of the addiction (number of years) and the severity of the cognitive bias.

**Conclusions:**

IGA present cognitive biases towards information related to Internet gaming. These biases, as well as poor executive functioning skills (lower mental flexibility and response inhibition), might be responsible for Internet game addiction. The assessment of cognitive biases in IGA might provide a methodology for evaluation of therapeutic effects.

## Introduction

With the Internet’s rapid advance and social penetration, its negative effects have emerged prominently. Many research reports have indicated that some on-line users are becoming addicted to the Internet, in much the same way that other individuals become addicted to drugs, alcohol, or gambling, which results in academic failure, reduced work performance and even marital discord and separation [1]–[3]. Currently, Internet addiction disorder (IAD), also described as pathological Internet use or problematic Internet use, is defined as an individual’s inability to control his or her use of the Internet, which eventually causes psychological, social, school, and work difficulties or dysfunction in a person’s life [4]–[6]. The description of IAD is based on the definition for substance dependence or pathological gambling, which makes it a compulsive-impulsive spectrum disorder. Studies have reported that IAD consists of at least three subtypes: excessive gaming, sexual preoccupations, and e-mail/text messaging. All of the subtypes share the common components, i.e., preoccupation, mood modification, excessive use, withdrawal, tolerance and functional impairment [7]. By using the Diagnostic and Statistical Manual of Mental Disorders (Fourth Edition, DSM-IV) criteria, some authors suggest that IAD is an impulse disorder or that it is at least related to impulse control disorders [8]–[9]. One study investigated deficient inhibitory control in individuals with IAD using a visual go/no-go task by event-related potentials (ERPs). This study indicated that individuals with IAD were more impulsive than the controls and shared neuropsychological and ERPs characteristics of compulsive-impulsive spectrum disorder, which supports the conclusion that IAD is an impulse disorder or is at least related to impulse control disorders [10].

In many situations of everyday life, the environment changes quite rapidly, requiring flexible behavioural adaptations. Many previous studies indicated that the task-switching paradigm has been a powerful method to study flexible behavioural adaptations to changing contexts [11]. The field of cognitive psychology has been interested in the use of this methodology, mainly because of factors that can potentially affect adaptive behaviour. These factors include the time participants have to prepare for a new task as well as response-related processes, such as response selection and execution. Many compulsive-impulsive spectrum disorders present cognitive bias and executive functional deficit characteristics. For example, using the “Alcohol Shifting Task”, a variant of the go/no-go paradigm, Xavier Noël and colleagues measured the response times and the accuracy of responses to targets and distracters [12]. Sometimes the alcohol-related words were the targets for the “go” response, with neutral words used as distracters, and sometimes the reverse scenario was presented. Several shifts in the type of the target occurred during the task. Relative to controls, the detoxified polysubstance abusers with alcoholism were generally slower to respond to targets. A signal detection analysis also indicated that, relative to controls, the detoxified polysubstance abusers with alcoholism had increased difficulty discriminating between targets and distracters, and they showed more signs of decision bias, reflecting an increased readiness to respond to both targets and distracters. However, these discrimination and inhibition deficits were more pronounced when alcohol-related words were the targets. These results suggest that detoxified polysubstance abusers with alcoholism have cognitive biases towards information related to alcohol and that these biases, as well as poor executive functions (lower mental flexibility and response inhibition), might be responsible for the failure of these individuals to maintain abstinence.

Another study examined the relationship between attention and gambling behaviour by measuring the level of Stroop interference towards gambling-related words in a group of regular poker machine players [13]. A computerised gambling-specific modified version of the Stroop task was used to assess response latencies. The test included three word categories: gambling, drug and neutral. The study found that the participants who had difficulty in controlling their gambling behaviour (the low control group) took significantly longer to name the colour of the words related to gambling, whereas those who had good control over their gambling behaviour (the high control group) did not show any significant differences among the three word categories. These results showed the role of cognitive distortions and biases in addictive gambling behaviour.

Recently, a research study investigated cognitive biases among pathological gambling poker players, experienced poker players and inexperienced poker players in a computerised two-player poker task that used a fictive opponent [14]. The results showed that experienced poker players had a significantly lower average margin of error in estimation of probability than pathological gambling poker players and inexperienced poker players and that pathological gambling poker players played hands with lower winning probabilities than inexperienced poker players. The study concluded that pathological gambling players presented with impaired cognitive bias styles related to probability estimation and decision making. These impairments may have implications for the assessment and treatment of cognitive biases in pathological gambling poker players.

The task-switching paradigm, such as the cue-related go/no-go switching task, provides an experimental approach to study this flexibility in changing situations [12]. Because IAD belongs to the compulsive-impulsive spectrum disorder, theoretically, it should present with the cognitive bias and executive functioning deficit characteristics of some disorders, such as pathological gambling, drug addiction or alcohol abuse, in testing with the cue-related go/no-go switching task. Until now, no studies on cognitive bias and executive function involving mental flexibility and response inhibition in IAD have been reported. Because Internet game addiction is a type of IAD, we selected individuals with Internet game addiction (IGA) as research subjects in this study. Participants’ behavioural responses were recorded while they performed an Internet game-shifting task. The purpose of the present study was to examine whether IGA displays cognitive bias and executive functioning deficit characteristics in an Internet game-shifting task.

## Materials and Methods

All research procedures were approved by the Ethics Committee on Human Studies of Nanjing Medical University (China) and were conducted in accordance with the Declaration of Helsinki. All participants gave written informed consent to participate in this study.

### 2.1 Diagnostic Approaches and Subjects

The criteria for selection of the IGA group were as follows: a) met the criteria of the modified Diagnostic Questionnaire for Internet Addiction (YDQ) (Appendix S1 [15], where subjects who answered “yes” to questions 1 through 5 and at least one of the remaining three questions were classified as suffering from IAD; b) aged greater than 18 years old; c) did not meet criteria of any DSM-IV axis I disorder or personality disorders by administering a structured clinical interview (Chinese version); d) did not have a diagnosis of alcohol or substance dependence, neurological disorders, any kind of head injury or systemic disease that might affect the central nervous system; and e) were not smokers. Additionally, to detect cognitive biases towards Internet game-related stimuli and to eliminate the effect of habituation when performing the trial, the subjects were Internet game addicts who had never played the Internet game “World of Warcraft”.

The duration of the disease was estimated via a retrospective diagnosis. We asked the subjects to recall their lifestyle when they were initially addicted to the Internet. To ensure that they were suffering from Internet addiction, the subjects’ recollection of past behaviour was retested with the criteria of the modified YDQ. We also confirmed the reliability of these self-reports by talking with the subjects’ parents via telephone. The IAD subjects spent 11.05±1.63 hours per day on online gaming. The number of days of Internet use per week was 6.34±0.5. We also verified this information from the roommates and classmates of the IAD subjects, finding that they often insisted on being on the Internet late at night, continuing to disrupt others’ lives despite the consequences. We conducted this study from May 1st, 2009, to January 26th, 2012. The subjects were recruited from the Internet Addiction Disorder Therapeutic Department of Wuxi Mental Health Center. A total of 46 subjects were recruited to form the IGA group. The control subjects (Non-Internet Addiction Disorder, NIA) were recruited from citizens living in Wuxi City, Jiangsu Province, China, through local advertisements.

Controls were excluded from the study if they were smokers or had a diagnosis of alcohol or substance dependence, neurological disorders, or any kind of head injury or systemic disease that might affect the central nervous system. Forty-six healthy persons matched to the test group by age, gender and education were recruited as the control group. According to a previous IAD study [16], we chose healthy controls who spent less than 2 hours per day on the Internet. The controls were also tested with the YDQ criteria modified by Beard and Wolf to ensure that they were not suffering from IAD. All participants were Chinese. All participants underwent a clinical assessment by a psychiatrist to collect information on medication and socio-demographic data and to confirm or exclude an IGA diagnosis. Handedness was assessed using the Annett handedness scale [17].

The demographic characteristics of the sample are detailed in Table 1.

**Table 1:** Demographic characteristics of the sample.

	IGA group	NIA group
Sex ratio (M/F)	46 (32∶14)	46 (32∶14)
Mean age (SD)	26(8)	26(8)
Age range in years	18–36	18–36
Internet game types (%)		
*Love Beat*	*15(33)*	–
*Audition*	*14(30)*	–
*Handgame Tetris*	*14(30)*	–
*Counter-strike*	*14 (30)*	–
*Maple Story*	*13(28)*	–
*Braid*	*12(26)*	–
*Little Big Planet*	*12(26)*	–
*Rock Band 2*	*12(26)*	–
*Gears of War 2*	*12(26)*	–
*Dead Space*	*11(24)*	–
*Hunted Forever*	*10(22)*	–
*Spore*	*9(20)*	–
*Fieldrunner*	*9(20)*	–
*Star Wars*	*7(15)*	–
*The Tower of Eternity*	*6(13)*	–
*Fallout 3*	*6(13)*	–
*Lunatia*	*6(13)*	–
*CS Online*	*5(11)*	–
*Aika Online*	*5(11)*	–
*Slugger*	*4 (9)*	–
*City Racer*	*4 (9)*	–
*Warrock*	*3 (7)*	–
*Metal Rage*	*3 (7)*	–
*Gunz*	*2 (4)*	–
*Keroro Pangpang*	*2 (4)*	–
Education (SD)	9(4)	9(4)
Years of addiction (SD)	3(2)	–
Hours per week of playing Internet games	46(8)	5(3)

M: male. F: female. SD: standard deviation.

### 2.2 Internet Game-shifting Task

**Figure 1:**
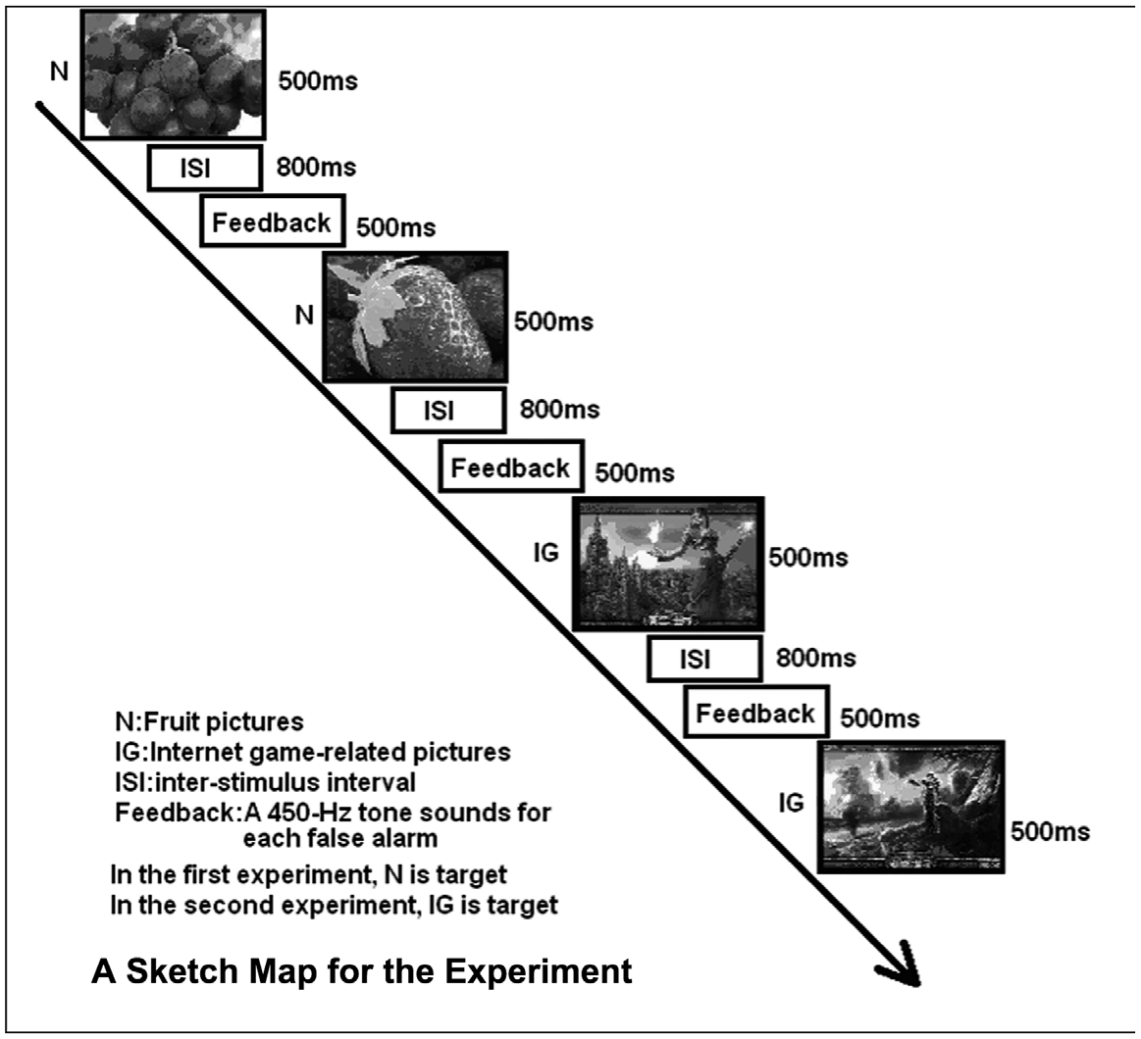
Internet game-shifting task.

E-Prime software (Edition, 2.0. Psychology Software Tools Inc., Sharpsburg, North Carolina, USA) was used for the trial procedure. The Internet game-shifting task, referred from Noël et al [12]. In the task, 10 pictures were selected from the Internet game “World of Warcraft” and 10 pictures were fruits. The pictures were briefly displayed, one by one, in the centre of the screen. There were two experiments in the task. In the first experiment, the Internet game “World of Warcraft” pictures were the targets and the fruit pictures were the distracters. In the second experiment, the fruit pictures were the targets and the Internet game “World of Warcraft” pictures were the distracters. Before the experimental trial began, instructions were displayed on the screen. Subjects were instructed to respond to targets by pressing the space bar as quickly as possible but not respond to the distracters. The pictures were presented for 500 ms, with an interstimulus interval of 800 ms. A 500-ms/450-Hz tone sounded for each false alarm (i.e., a response to a distracter), but no tone sounded for omissions (i.e., failures to respond to a target). The task was comprised of 2 practice blocks followed by eight test blocks. Each block of 20 stimuli was composed of 10 fruit pictures (Neutral Pictures, N) and 10 Internet game-related pictures (IG). In each block, either N or IG pictures were specified as targets, with targets for the ten blocks presented either in the order N N IG IG N N IG IG N N or IG IG N N IG IG N N IG IG. Due to this arrangement, four test blocks were ‘non-shift’ blocks, where subjects must continue responding to stimuli in the same way. Four test blocks, however, were ‘shift’ blocks, where subjects must begin responding to stimuli, which had been distracters, and cease responding to stimuli, which had been targets (Figure 1).

### 2.3 Statistical Analyses

The data were analysed using SPSS (version 10.0 International Business Machines Corporation, New York, USA). The hit rate and reaction time (RT) to a target were used as the primary dependent measures. A signal detection analysis was performed to determine discrimination (*d*′). According to previous research [18], the formulas used for signal detection analysis are as follows: 1) Hit probability = (∑ answer)/(∑ target); 2) False alarm probability = (∑ answer)/(∑ non-target); 3) Corrected hit probability = (∑ answer +0.5)/(∑ target +1); 4) Corrected false alarm probability = (∑ answer +0.5)/(∑ non-target +1); 5) *d′* = Z(Corrected hit probability) – Z(Corrected false alarm probability); 6) C = −0.5 × [Z (Corrected hit probability)] + Z (Corrected false alarm probability)]. In the above formulas, ‘∑ answer’ is the total number of responses, ‘∑ target’ is the total number of targets, and ‘∑ non-target’ is the total number of distracters. Hit probability is the probability to respond to a target. False alarm probability is the probability of responding to a distracter. Z(*p*) is the quantile function of the normal distribution. Z-values are calculated on corrected probability to avoid obtaining an infinite value when P = 1.

A group (IGA versus NIA, between)×target (IG versus neutral, within)×condition (shift versus no-shift, within) analysis of variance (ANOVA) was performed using the hit rate, RT, d*′* and C variables. Cognitive biases were inferred from the group–target interactions. Shifting abilities were inferred from the group–condition interactions. Using the method of subtracting the value to shift from the value to non-shift as assessment measure of the switch effect for hit rate, RT, *d′* and C respectively; Using the method of subtracting the value to neutral from the value to Internet-game as assessment measure of the target effect for hit rate, RT, *d′* and C respectively.

## Results

### 3.1 Comparisons of Behaviour Data

#### 3.1.1 Internet game-shifting task: Accuracy rate

**Figure 2:**
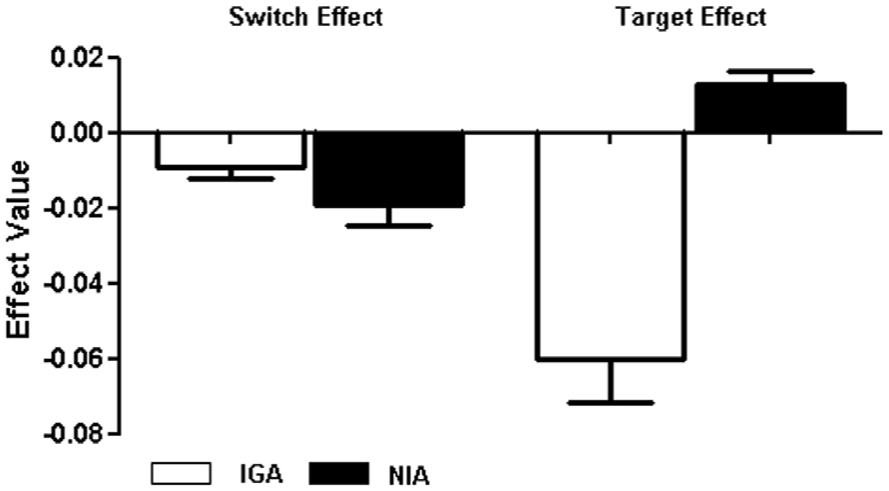
The switch effect and target effect value and standard-error bars for hit rate between IGA (white) and NIA (black) group on the Internet game-shifting task.

Using hit rate as the dependent measure, a three-way ANOVA (group×target×condition) revealed a significant effect from group type (*F*_1,90_ = 5.93, *P* = 0.014), with IGA having lower hit rate than NIA. This main effect was driven by the IGA group, as evidenced by a group-target interaction. The group–target interaction effect was significant (*F*_1, 90_ = 6.32, *P* = 0.021). It revealed a significant effect from the type of target (*F*_1,90_ = 9.17, *P* = 0.015), with IGA having higher hit rate when the targets were Internet game-related pictures as opposed to neutral pictures. In addition, an effect of condition was identified (*F*_1, 90_ = 6.25, *P* = 0.035), with a higher hit rate in the non-shift condition (Table 2). The switch effect and target effect value and standard-error bars for hit rate represented as Figure 2.

**Table 2:** Mean Accuracy rate (Hit & False alarm), RTs d*′* and C for IGA and NIA group on the Internet game-shifting task (mean ± SD).

	IGA group (n = 46)		NIA group (n = 46)	
*Accuracy rate*	*Hit rates*	*False alarm* *rates*	*Hit rates*	*False alarm rates*
*Condition*				
Non-shift	0.91±0.08	0.08±0.02	0.92±0.07	0.07±0.03
Shift	0.90±0.11	0.09±0.01	0.91±0.08	0.08±0.02
*Type of target*				
Neutral	0.90±0.10	0.11±0.09	0.91±0.08	0.07±0.03
Internet-game	0.96±0.04	0.04±0.05	0.91±0.09	0.08±0.01
***RTs***				
*Condition*				
Non-shift	534.95±35.40		499.67±26.90	
Shift	550.40±18.91		489.24±15.65	
*Type of target*				
Neutral	579.35±20.40		505.89±16.52	
Internet-game	517.87±19.18		481.75±24.31	
***d′***				
*Condition*				
Non-shift	2.17±0.94		2.56±0.74	
Shift	1.97±0.84		2.28±0.38	
*Type of target*				
Neutral	1.91±0.99		2.25±0.48	
Internet-game	2.10±0.83		2.49±0.34	
***C***				
*Condition*				
Non-shift	−0.21±0.31		−0.16±0.29	
Shift	−0.28±0.45		−0.16±0.33	
*Type of target*				
Neutral	−0.29±0.37		−0.26±0.34	
Internet-game	−0.24±0.39		−0.08±0.30	

#### 3.1.2 Internet game-shifting task: RT

**Figure 3:**
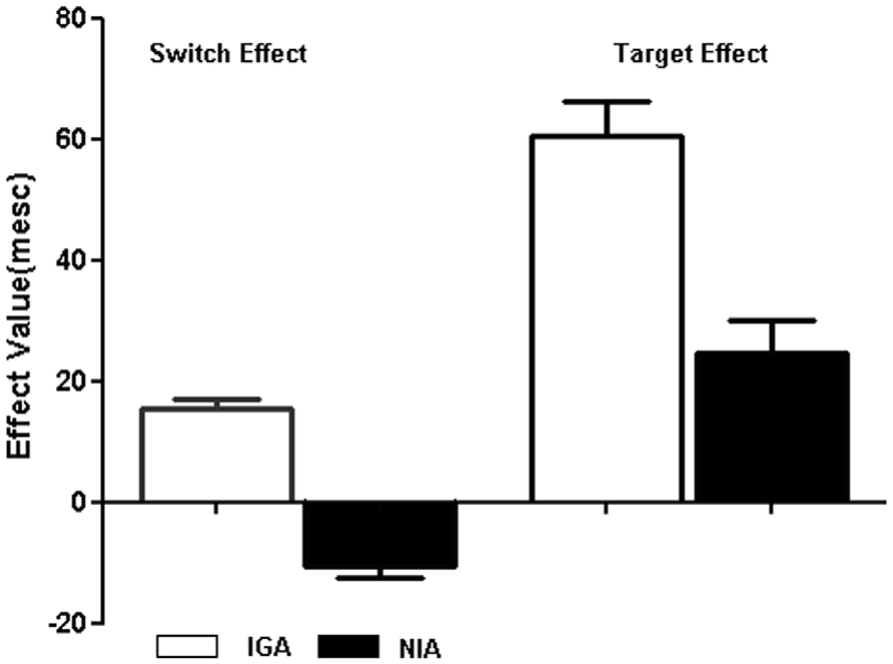
The switch effect and target effect value and standard-error bars for RTs between IGA (white) and NIA (black) group on the Internet game-shifting task.

Using RT as the dependent measure, a three-way ANOVA (group×target×condition) revealed a significant effect from group type (*F*_1,90_ = 3.15, *P* = 0.024), with IGA being slower than NIA in their processing speed when detecting targets. An effect from type of target also emerged from this analysis (*F*_1, 90_ = 7.15, *P* = 0.016), with participants being slower to detect neutral targets than Internet game-related targets. The group–target interaction effect was significant (*F*_1, 90_ = 15.24, *P* = 0.011). Comparisons of the means revealed that the slowing down of IGA, relative to NIA, was more pronounced when the target stimuli were neutral as opposed to Internet game-related pictures. In addition, the group–condition interaction effect was significant (*F*_1,90_ = 9.30, *P* = 0.013). Comparisons of these means indicated that the slowing down of IGA, relative to NIA, was more pronounced in the shift rather than the non-shift conditions (Table 2). The switch effect and target effect value and standard-error bars for RTs represented as Figure 3.

#### 3.1.3 Internet game-shifting task: Discrimination (d′)

**Figure 4:**
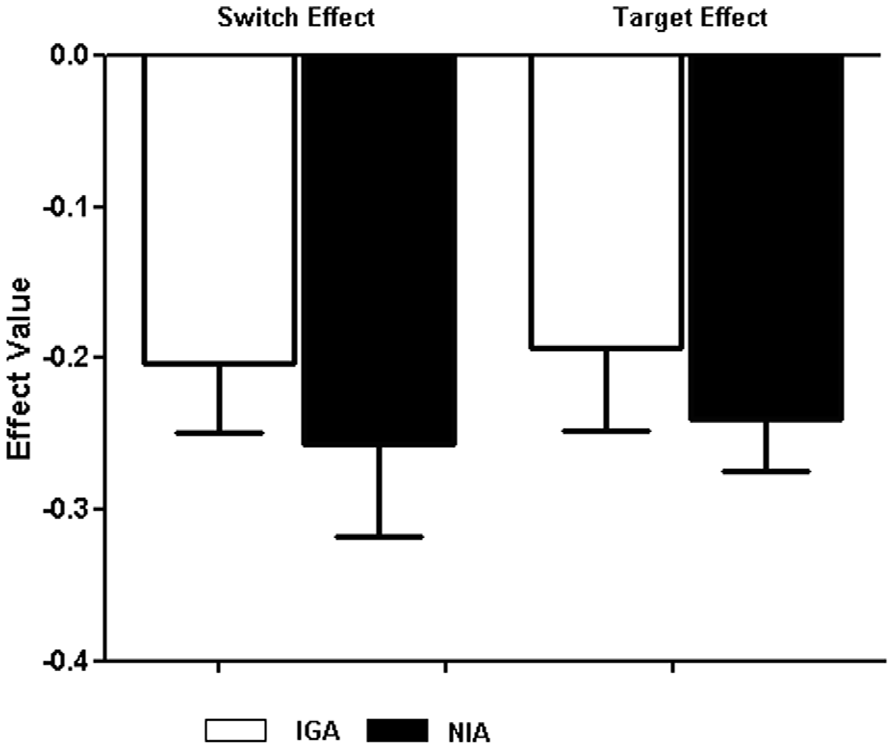
The switch effect and target effect value and standard-error bars for *d′* between IGA (white) and NIA (black) group on the Internet game-shifting task.

Using *d′* as the dependent measure, a three-way ANOVA (group×target×condition) revealed a main effect from group type (*F*_1, 90_ = 6.21, *P* = 0.019), with IGA having lower discrimination ability than NIA. This main effect was driven by the IGA group, as evidenced by a group-target interaction. The group–target interaction effect was significant (*F*_1, 90_ = 4.16, *P* = 0.037). It revealed a significant effect of type of target (*F*_1,90_ = 6.17, *P* = 0.018), with IGA having a higher discrimination ability when the targets were Internet game-related pictures than NIA. In addition, an effect of condition was found (*F*_1, 90_ = 5.35, *P* = 0.031), with discrimination being better in the non-shift condition. Comparisons of the means revealed that the impaired discrimination of IGA was more pronounced when the targets were Internet game-related pictures rather than neutral pictures. These results are presented in Table 2. The switch effect and target effect value and standard-error bars for *d′* represented as Figure 4.

#### 3.1.4 Internet game-shifting task: Decision bias (C)

**Figure 5:**
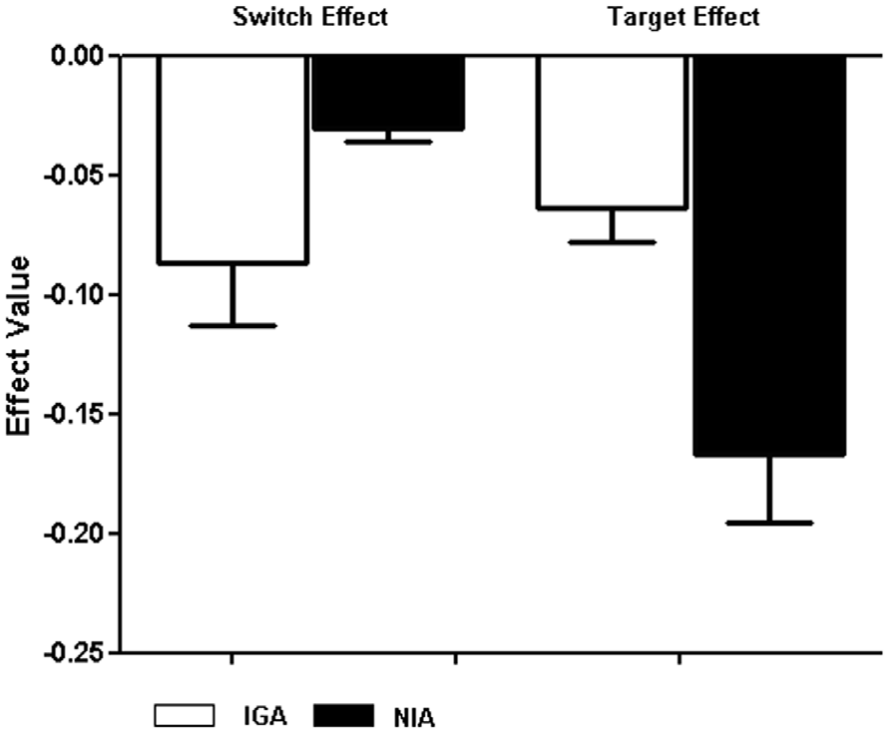
The switch effect and target effect value and standard-error bars for C between IGA (white) and NIA (black) group on the Internet game-shifting task.

Using C as the dependent measure, a three-way ANOVA (group×target×condition) showed that group type produced a significant effect, with IGA expressing more decision bias than NIA (low C).(*F*_1, 90_ = 8.24, *P* = 0.017. It also revealed an effect from type of target (*F*_1, 90_ = 15.74, *P* = 0.005), with all participants displaying a weaker decision bias when the targets were Internet game-related pictures (high C). A significant effect of condition was also observed (*F*_1, 90_ = 8.31, *P* = 0.018), with disinhibition being stronger in the shift condition. The group–target interaction effect was significant, with IGA having a stronger decision bias than NIA (low C), especially when the targets were Internet game-related pictures. (*F*_1, 90_ = 9.79, *P* = 0.012). The group–condition interaction effect was significant, with IGA having a stronger decision bias in the shift condition (low C) (*F*_1, 90_ = 9.89, *P* = 0.011). These results are presented in Table 2. The switch effect and target effect value and standard-error bars for C represented as Figure 5.

### 3.2 Relationship between Years of Addiction and Cognitive Measures

**Figure 6:**
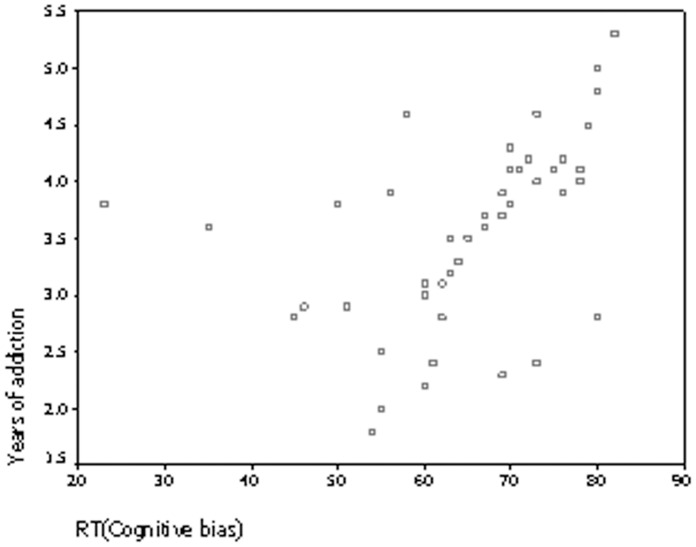
Relationship between years of addiction and cognitive measures in IGA.

Using the method of subtracting the RT to neutral pictures from the RT to Internet game-related pictures as an assessment measure of cognitive bias, there was a positive correlation between the years of addiction and the cognitive bias (*r = *0.38, *P* = 0.012). (Figure 6). The correlation corrected for age of IAG group is 0.11.

## Discussion

This study is the first to employ the Internet game-shifting task in investigating cognitive bias and executive functioning deficit characteristics in individuals with Internet game addiction. In this study, we measured the mechanism of executive control by response inhibition and shifting and the cognitive bias for Internet game-related pictures. Our trial results showed that individuals with IGA exhibit response inhibition and shifting deficits and cognitive biases towards Internet game-related stimuli.

On our modified version of a go/no-go paradigm (i.e., Internet game-shifting task), a higher number of false alarms (response to a distracter) combined with a lower number of hits (response to a target) indicate an inability to discriminate targets from distracters. False alarms alone cannot be interpreted as an indication of disinhibition. Therefore, we performed a signal detection analysis to distinguish d′ from C.

We used the time taken to respond to a target (RT) as the primary dependent measure and performed a signal detection analysis to distinguish d′ from C. In our study, a group×target×condition ANOVA was performed on RT, d′ and C. For hit rate, RT and d′, the group–target interaction effect was significant. Because cognitive biases are inferred from the group–target interactions, our results indicated that IGA presented cognitive biases towards Internet game-related stimuli. Additionally, in comparison with matched NIA, IGA took longer to respond to targets, especially when the targets were neutral pictures. This effect was greater when there was a shift in targets from Internet game-related pictures to neutral pictures or vice versa. Reaction time analyses suggested that IGA allocated more attentional resources to Internet game-related pictures than to neutral pictures. Our results showed that for RT, the group–condition interaction effect was significant. Because shifting abilities are inferred from the group–condition interactions, comparisons of these means indicated that the slowing down of IGA, relative to NIA, was more pronounced in the shift than in the non-shift conditions.

A d′ value of 0 or less indicates that subjects were unable to discriminate targets from distracters or were not performing the task as instructed. A high d′ indicates good discrimination ability (i.e., more hits and less false alarms). In this study, using d′ as the dependent measure, the results revealed a main effect from group type, with IGA having lower discrimination ability than NIA. The results also revealed a significant effect of type of target, with IGA having higher discrimination ability when the targets were Internet game-related pictures than NIA. In addition, an effect of condition was found, with discrimination being better in the non-shift condition than in the shift condition.

In signal detection theory, C being less than 0 reflects a higher readiness to respond to any stimulus, for example, more hits and more false alarms. When C is greater than 0, this indicates a decreased tendency to respond to any stimulus, i.e., less hits and less false alarms. Because C takes into account both hits and false alarms, it is a better indicator of disinhibition than false alarms alone. Thus, a low C was considered a sign of disinhibition. In this study, the NIA group shows a larger target effect in C than that of the IGA group, which deduces that the IGA group displays more disinhibition in general relative to the NIA group.

Taking decision bias as a sign of disinhibition, our results of the signal detection analysis indicated that IGA expressed more decision bias than NIA (low C), all participants displayed a weaker decision bias when the targets were Internet game-related pictures (high C), and disinhibition was stronger in the shift condition. The group–target interaction effect was significant, indicating that IGA had a stronger decision bias than NIA (low C), especially when the targets were Internet game-related pictures. The group–condition interaction effect was significant, indicating that IGA have a stronger decision bias in the shift condition (low C).

Our results are similar to the study of cognitive biases for alcohol-related cues on executive function tasks involving mental flexibility and response inhibition in polysubstance abusers with alcoholism [12]. Consistent with previous research [10], [19], our study determined that impaired executive control in IAD supports that IAD might be an impulse disorder, or at least is related to impulse control disorder, just as alcohol dependence is indirectly related.

As for individuals with drug addiction and alcohol dependence, the presence of a cognitive attentional bias for drug or alcohol cues is congruent with both the psychopharmacological and neuro-anatomical perspectives. When conditioned drug stimuli are present, increased dopamine levels in the corticostriatal circuit, particularly in the anterior cingulate, amygdala and nucleus accumbens, serves to draw the subject’s attention towards the drug related stimulus [20]. Future research should confirm whether IGA presents this physical characteristic.

In our study, there was a positive correlation between the duration of Internet game addiction and attentional bias for Internet game-related cues, indicating that this cognitive bias may play a role in the severity of IGA. Individuals with IAD present mental health problems related to excessive computer use. These issues may include fear and anxiety related to withdrawal, avoidance of social interaction other than through Internet sources, withdrawal from family and personal relationships, and deviant behaviour. From a treatment perspective, a general approach of inclusion of the symptoms of Internet addiction within the larger context of psychotherapy was used [9]. One key to intervention is the motivation to change, which can be particularly problematic with the current reliance on the Internet for a majority of work and leisure activities [21]. The implications of this study are that the assessment of cognitive biases in IGA might provide a methodology for evaluation of therapeutic effect.

The research results of this study are preliminary because of the small sample size. Further studies with larger sample sizes are needed to replicate our findings. In addition, this study used YDQ scores of higher than 6 as an indicator of IAD. Although this questionnaire is a frequently used instrument for assessing IAD, its validity as a diagnostic instrument has been questioned [22]. Future studies may utilise other measures of assessing diagnostic criteria or severity of Internet addiction problems to assess the relationship between cognitive bias and executive functioning deficit in IAD.

### 4.1 Conclusion

Based on our data, we conclude that IGA have cognitive biases towards information related to Internet games. IGA presents with the cognitive bias and executive functioning deficit characteristics of some compulsive-impulsive disorders, such as pathological gambling, drug addiction or alcohol abuse. These cognitive biases, as well as poor executive functioning skills (lower mental flexibility and response inhibition), might be responsible for Internet addiction disorders.

## Supporting Information

Appendix S1 **Young’s Diagnostic Questionnaire for Internet Addiction (YDQ).**
(DOC)
