# Supplementary material for: Cognitive Biases toward Internet Game-Related Pictures and Executive Deficits in Individuals with an Internet Game Addiction
Source: PLoS One. 2012 Nov 14;7(11):e48961. doi: 10.1371/journal.pone.0048961 (PMC3498351; doi:10.1371/journal.pone.0048961)
Supplement: Appendix S1 — Young’s Diagnostic Questionnaire for Internet Addiction (YDQ). (DOC) [file pone.0048961.s001.doc]

**Supporting Information**

**Appendix S1**

Young’s Diagnostic Questionnaire for Internet Addiction (YDQ)

1. Do you feel preoccupied with the Internet (e.g., thinking about previous online activity or anticipating your next online session)?

2. Do you feel the need to use the Internet with increasing amounts of time in order to achieve satisfaction?

3. Have you repeatedly made unsuccessful efforts to control, cut back, or stop Internet use?

4. Do you feel restless, moody, depressed, or irritable when attempting to cut down or stop Internet use?

5. Do you stay online longer than originally intended?

6. Have you jeopardised or risked the loss of significant relationship, job, educational, or career opportunity because of the Internet?

7. Have you lied to family members, therapists, or others to conceal the extent of your involvement with the Internet?

8. Do you use the Internet as a way of escaping from problems or of relieving a dysphoric mood (e.g., feelings of helplessness, guilt, anxiety, or depression)?
